# Delayed Saddle Cement Pulmonary Embolism 15 Years After Kyphoplasty

**DOI:** 10.51894/001c.165972

**Published:** 2026-07-31

**Authors:** Mohamed Mukhtar, Griffin Dufek, Reginald Sandy

**Affiliations:** 1 Henry Ford - Genesys

## 107

### Background

Pulmonary cement embolism is a recognized but uncommon complication of vertebral augmentation in which polymethylmethacrylate cement migrates into the pulmonary arterial circulation. Reported radiographic incidence ranges from approximately 2% to 26% depending on screening practices, while symptomatic and centrally located emboli are rare, reported in well under 1% of cases, and lack standardized management guidance.

### Case Description

A patient with asthma, hypertension, osteopenia, and a 49-pack-year smoking history (quit 1 year prior) with prior spinal fusion and kyphoplasty presented with 2 weeks of fatigue, presyncope, and pleuritic chest pain with acute worsening and left arm radiation. CT pulmonary angiography showed linear hyperdensities in the main pulmonary artery and right and left distal interlobar segmental branches consistent with vertebroplasty cement emboli, with multilevel vertebroplasty from T7–T11. Intravenous heparin infusion was initiated on admission after CTA diagnosis. Transthoracic echocardiography, cardiac biomarkers, and hemodynamics were assessed. Percutaneous mechanical thrombectomy using a FlowTriever system targeted the right basilar pulmonary artery, right truncus anterior, and left basilar pulmonary artery with attempted foreign body retrieval.

### Results

Echocardiography showed left ventricular ejection fraction 55–60% with normal right ventricular size and systolic function and no right ventricular strain. Troponin was <0.01 on two measurements and BNP was <10. The patient remained normotensive with systolic blood pressure in the 120s–140s and heart rate in the 80s without hemodynamic compromise. Thrombectomy was attempted but cement fragments could not be removed. The patient was transitioned at discharge to apixaban 10 mg twice daily for 14 days followed by 5 mg twice daily. The patient remained on room air, was monitored on telemetry, and hospitalized for 3 days. Symptoms improved and the patient was discharged in stable condition. Two-week follow-up showed continued stability. Hypercoagulable testing is pending and follow-up CTA is planned.

### Conclusions

Pulmonary cement embolism is a rare but clinically important complication of vertebral augmentation that may present years after the procedure. This case highlights characteristic CTA findings, technical limitations of endovascular cement retrieval, and the role of anticoagulation with close follow-up in a stable patient.

